# The journal: one year later

**DOI:** 10.1186/1749-7221-2-20

**Published:** 2007-09-28

**Authors:** Rahul K Nath

**Affiliations:** 1 Texas Nerve & Paralysis Institute, Houston, Texas, USA

## Abstract

This article celebrates the first year anniversary of the *Journal of Brachial Plexus and Peripheral Nerve Injury*.

## Editorial

As Editor-in-Chief, it is my pleasant duty to celebrate the first year anniversary publication of the *Journal of Brachial Plexus and Peripheral Nerve Injury*. Only a year has passed since the inaugural issue and many significant events have occurred in this brief time. We have surpassed expectations as far as quality and breadth of submitted articles and we are setting the standard for publications in this field.

I believe that in the past year we have published more scientific articles about the brachial plexus than any other journal. This is a critical point as it signifies that the scientific community is accepting our journal as an important outlet for brachial plexus research. To have this large volume in such a short time also validates the underlying premise behind developing the journal, namely that there was an unmet need for a specialty academic publication. I believe that we have met that need.

There have been many interesting submissions, and they have come from all corners of the globe. Countries that have been represented are: The Republic of South Africa, India, Cuba, Iran, Colombia, Japan, Brazil, Slovenia, Germany, The United States, Italy, Greece, Egypt, Australia, and Turkey.

In addition to standard scientific format, we have also published video findings from Professor Roman Bošnjak (additional file 1, Bošnjak et al. [1]) and Powerpoint presentations sent by Professor Sayed Rayegani from the Iranian Congress of Electrodiagnosis (additional file 1, Rayegani et al. [2]). Both of these are exciting formats that allow in-depth presentation of complex ideas in ways that enhance our insight. I think that the use of video and other visual techniques will be increasingly important in presenting comprehensive descriptions of new concepts to our audience, and as an online publication *Journal of Brachial Plexus and Peripheral Nerve Injury *is ideally suited to publish movies and other image files. I am very pleased that our initial forays into the use of these new media have been so successful.

Our published authors have included major figures in all fields of brachial plexus and peripheral nerve injury, as well as rising stars. We have been privileged to learn about new surgical techniques [3], new diagnostic measures [4], and new basic science findings [5] that may all have immediate impact in our practices and scientific endeavors. I would not hesitate to say that every one of our published articles has given me much to think about and that every one has enhanced my appreciation of this fertile area of science.

Three of our articles have been designated as "Highly Accessed" [6-8] and one, by Professor Marianna Papadopoulou and colleagues [8], has been downloaded over 5,000 times from the journal, meaning that it has been viewed over 10,000 times when PubMed Central accesses are included (each of our articles is indexed in PubMed and archived in PubMed, as well as in other freely accessible repositories [9], imediately upon acceptance, and it is safe to assume that the readership for a particular article is twice as high as the count for accesses to the journal's homepage directly). It is noteworthy that this readership finds our articles by searching for specific topics of interest, so that we are reaching those who can most appreciate and find use for published information.

The mix of basic science and clinical materials submitted for review has been excellent and encouraging. Our reviewers are all voluntary and I must thank them for lending us their invaluable expertise and, more significantly, their time. The act of assisting in this way truly shows a level of commitment and caring that is difficult to celebrate adequately. I would also like at this moment to thank Sonya Melcher for her excellent management of the journal. Sonya has always been diligent and impartial, two requisite characteristics for any managing editor.

This is also an appropriate time to acknowledge the friendship and the contributions of Professor Rolfe Birch who has recently retired from full-time surgical and academic practice. As such, he remains involved with the journal as Emeritus Editor-in-Chief as well as with the International Society of Brachial Plexus and Peripheral Nerve Injury [10], still there for us when we need his advice and insight. Thank you, Professor Birch, for your mentorship and calming influence at the beginning. We will continue to rely on you as we go forward.

As we look to the coming year, I remain positive about the direction of the journal and the Society. We have more support than ever with the inclusion of new editors and new ideas. We have added Jörg Bahm and Jayme Bertelli as Deputy Editors and several other colleagues, including Lee Dellon, to the Editorial Board. I anticipate significant growth in our readership as well as our editorial content. We are aiming to be tracked by Thomson Scientific to receive an impact factor as soon as possible. As an additional service, we have added a new feature to the journal's homepage, "Latest News," which presents upcoming relevant scientific symposia of interest to our readership. Any meeting coordinator who wishes to include their conference should contact the Managing Editor.

In conclusion, I would like to thank everyone who has been involved with the journal during the planning, startup and running phases. Every expectation has been exceeded and I look forward to increasing the scope and number of published articles in the future. Our editorial board has steadily grown to include leaders in our fields of interest as well as new members of our discipline. The underlying International Society of Brachial Plexus and Peripheral Nerve Injury has also grown to include about 150 members, a significant number for what is still a relatively small specialty, another positive sign for future development of both the Society and the journal. We will be planning the next meeting of the Society in 2008 and will make announcements as appropriate. The staff at BioMed Central continue to support the activities of the journal in a professional and collegial manner. This allows us to continue to improve our scientific services to our readers. We look forward to many more years of partnership.

Thank you again to all our contributors. In simply continuing the commitment to excellence that has made you leaders, – the journal and Society will necessarily grow and prosper.
